# Machine Learning Algorithms for Classification of MALDI-TOF MS Spectra from Phylogenetically Closely Related Species *Brucella melitensis*, *Brucella abortus* and *Brucella suis*

**DOI:** 10.3390/microorganisms10081658

**Published:** 2022-08-17

**Authors:** Flavia Dematheis, Mathias C. Walter, Daniel Lang, Markus Antwerpen, Holger C. Scholz, Marie-Theres Pfalzgraf, Enrico Mantel, Christin Hinz, Roman Wölfel, Sabine Zange

**Affiliations:** 1 Bundeswehr Institute of Microbiology, Neuherbergstrasse 11, 80937 Munich, Germany; 2 Robert Koch Institut (RKI), Centre for Biological Threats and Special Pathogens, Seestr. 10, 13353 Berlin, Germany

**Keywords:** MALDI-TOF MS, *Brucella melitensis*, *B. suis*, *B. abortus*, machine learning, nested *k*-fold cross validation, feature selection, R

## Abstract

(1) Background: MALDI-TOF mass spectrometry (MS) is the gold standard for microbial fingerprinting, however, for phylogenetically closely related species, the resolution power drops down to the genus level. In this study, we analyzed MALDI-TOF spectra from 44 strains of *B. melitensis*, *B. suis* and *B. abortus* to identify the optimal classification method within popular supervised and unsupervised machine learning (ML) algorithms. (2) Methods: A consensus feature selection strategy was applied to pinpoint from among the 500 MS features those that yielded the best ML model and that may play a role in species differentiation. Unsupervised *k*-means and hierarchical agglomerative clustering were evaluated using the silhouette coefficient, while the supervised classifiers Random Forest, Support Vector Machine, Neural Network, and Multinomial Logistic Regression were explored in a fine-tuning manner using nested *k*-fold cross validation (CV) with a feature reduction step between the two CV loops. (3) Results: Sixteen differentially expressed peaks were identified and used to feed ML classifiers. Unsupervised and optimized supervised models displayed excellent predictive performances with 100% accuracy. The suitability of the consensus feature selection strategy for learning system accuracy was shown. (4) Conclusion: A meaningful ML approach is here introduced, to enhance *Brucella* spp. classification using MALDI-TOF MS data.

## 1. Introduction

The genus *Brucella* contains 12 species causing brucellosis in terrestrial and aquatic mammals. Significant clinical disorders in humans are mainly caused by *B. melitensis*, *B. abortus* and *B. suis*, in general upon the consumption of contaminated dairy products or the handling of infected animals. Although the mortality rate caused by this pathogen is low, *Brucella* spp. is a facultative intracellular pathogen that can escape recognition by innate immunity and evade intracellular destruction [1,2], leading to chronic infectious disease which is difficult to treat. Furthermore, *Brucella* spp. is considered a potential bioterrorism agent because species belonging to this genus are highly infective (10–100 bacteria can cause human infection), and can be easily cultured from infected animals and human materials, stored, and potentially disseminated through aerosol. Clearly, timely and accurate diagnostics is crucial for patient treatment decisions and outcomes, as well as pivotal for public health in case of outbreak or terrorist attack.

Matrix-assisted laser desorption/ionization time-of-flight (MALDI-TOF) mass spectrometry (MS) is a soft ionization proteomic platform, which is generally used in most clinical laboratories as a gold standard for microbial fingerprint-based characterization [3]. MALDI-TOF MS is a reproducible and reliable tool that allows bacterial identification within a few minutes, with low cost for a single reaction. However, for phylogenetically closely related species such as *B. melitensis*, *B. abortus* and *B. suis*, the resolution power drops down to the genus level [4]. This might be due to the absence of a comprehensive database, especially for biosafety level three species [4,5], and to the lack of appropriate methods for optimal species recognition.

Machine learning (ML) is a sub-field of artificial intelligence (AI) that gives computers the ability to learn without being explicitly programmed [6].

In the last decade, machine learning (ML) techniques have been recognized as a fundamental resource to build informative and predictive models from complex biological data [7].

In the field of microbiology, several studies have shown how the combination of ML algorithms with mass spectra information can enhance discrimination between closely related sublineages within the genera *Mycobacterium* [8] and *Bacillus* [9,10], as well as the identification of different antimicrobial resistant groups of *Staphylococcus aureus* [11,12].

Despite their great potential, the application of ML algorithms for bacterial species classification has been limited by the use of proprietary software such as flexAnalysis and ClinProTools from Bruker Daltonics (Bruker Daltonics GmbH and Co. KG, Bremen, Germany), where the applied pipelines are not accessible, severely affecting the comprehensibility, optimization, and reproducibility of analyses [13]. Furthermore, certain analytical and computational challenges have been encountered. The most relevant here is the high dimensionality of the MS data; even in relatively large studies with a few hundred samples, the number of variables often exceeds the number of samples themselves. Thus, to enable the construction of accurate classifiers requires a method to pinpoint biomarkers, called “feature selection”. For linear classifiers such as support vector machine or discriminant analysis, diagnosing and filtering out collinear variables (highly correlated predictor variables) is an essential step to avoid instability in the results generated by machine learning models [14]. A constant danger with ML classifiers is overfitting, where the classifier learns irrelevant information or “noise” from the training dataset. When this happen, the model becomes “overfitted” and is unable to make an accurate prediction or classification against new or unseen data. The most widely used way to prevent overfitting is by reducing model complexity (for example selecting only the most informative features), to apply regularization or hyperparameter optimization, employing cross-validation for model selection. Another potential pitfall when building a ML model is the class imbalance ratio. Class imbalance refers to an unequal number of cases for each sample category, for example, bacterial species. This can lead to failures in model accuracy, especially for minority classes, resulting in a higher error rate. To handle this type of data it is necessary to even the class imbalance by resampling the data, either by up-sampling the smaller classes or down-sampling the larger classes. Alternatively, an ML algorithm with built-in capabilities to help deal with imbalanced data may be employed. Nevertheless, facing a multiclass classification problem with more than two classes, a choice of algorithms developed to address such a problem should be considered, or strategies may be implemented to turn a natural binary algorithm such as logistic regression into a multinomial classifier.

The major aim of this study was to analyze MALDI-TOF MS spectra of 44 isolates of *Brucella*, including 21 *B. melitensis*, 12 *B. suis* and 11 *B. abortus,* in order to identify the most appropriate classification methods from among the popular supervised and unsupervised machine learning algorithms for optimal species recognition.

## 2. Materials and Methods

### 2.1. Study Design/Experimental Setup and Analysis

Three different *Brucella* species, identified by means of a PCR-based method, were analyzed using MALDI-TOF MS. The row MS spectra were pre-processed according to Gibb and Strimmer [15], and cleaned to create a peak matrix with sample ID and feature value intensities. To avoid the problem of dimensionality, a downstream consensus feature selection strategy was used to identify a reduced set of important features to feed all ML algorithms. To address the role of the consensus feature selection strategy in the accuracy of the learning systems, each ML algorithm was trained on the most important classifier-specific variables, and was compared with the models fitted on the consensus features. To avoid over-optimistic estimation of the model performance due to the small sample size and to avoid overfitting, all ML classifiers were optimized in terms of number of features and hyperparameters by means of a nested cross validation (nCV) method [16], with a feature reduction step between the two CV loops. For each final classifier, the expected performance or ability of the model to generalize well against new data was computed on all folds of the outer CV loop. The true performance was evaluated on an external test dataset, including 12 *Brucella* samples treated as unknown cases. All data analyses were completed in R (version: 3.6.3, R Core Team, 2013) and RStudio. A list of the R-packages used in this work is reported in Table S3.

### 2.2. Biological Sample Collection

All microbial strains used in this study are part of the strain collection of the Bundeswehr Institute of Microbiology and were stored at −80 °C in a Microbank microbial storage system (Pro-Lab Diagnostics, Richmond Hill, ON, Canada). In total, 21 clinical isolates of *Brucella melitensis*, 12 isolates of *B. suis* and 11 isolates of *B. abortus* from human or animal origin, were selected for spectra generation. The bacterial strains used for MALDI-TOF MS analysis and corresponding metadata are listed in Table S2. It should be noted that the dimensions of this study were defined by the total number of *B. suis* and *B. abortus* strains available in our laboratory. To avoid problems of class imbalance, a maximum of 21 *B. melitensis* strains were selected from among those that were been fully sequenced and characterized by Georgi et al. [17].

### 2.3. Bacterial Culture Preparation and Inactivation

Bacterial cultivation and inactivation were performed under biosafety level three (BSL-3) conditions in a class II biosafety cabinet. Bacterial strains were grown on Columbia blood agar plates at 36 °C with 5% CO_2_ for 48 h and sub-cultivated once under the same conditions. For DNA and protein extraction, two inactivated aliquots per sample were prepared by taking half of a 1 µL-loop of bacteria from the subcultivation plate, resuspending it in a solvent mixture containing 900 µL absolute ethanol and 300 µL double-distilled water, andincubating at room temperature for 5 min. Thereafter, the inactivated colony suspension was transferred to a BSL-2 laboratory for downstream analyses.

### 2.4. Brucella PCR-Based Methods

To confirm the species assignment of the strains, inactivated bacterial cells were washed twice with PBS, and genomic DNA was extracted using a QIAamp DNA Mini Kit (Qiagen, Hilden, Germany) according to the manufacturer’s instructions. *Brucella* genus was assessed by means of a polymerase chain reaction (PCR) targeting the genus-specific markers *IS711* and *bcsp31* [18]. For species differentiation, the *Brucella* Bruce-ladder PCR [19], a multiplex PCR with species-specific band pattern, was used. The species assignment of each isolate used in this study is reported in Table S1.

### 2.5. Protein Extraction and MALDI-TOF Spectrum Generation

Protein extracts were obtained from inactivated bacterial suspension according to the method described by Marklein et al. [20]. Briefly, the inactivated bacterial suspension was prepared for MALDI-TOF MS measurement by centrifuging at 13,000× *g* for 2 min and the supernatant removed. The step was repeated and the pellet allowed to dry, then resuspended in 50 µL of formic acid (70% *v*/*v*) and mixed thoroughly by pipetting. After 50 µL of acetonitrile (Sigma-Aldrich Chemie GmbH, Taufkirchen, Germany) was added, the mixture was vortexed and centrifuged at 13,000× *g* for 2 min. Then, 1 µL of each sample was spotted onto a 96-well steel target plate (Bruker Daltonics GmbH and Co. KG, Bremen, Germany) and overlaid with 1 µL of α-Cyano-4-hydroxycinnamic acid (HCCA) matrix. The matrix solution was prepared by dissolving 2.5 ± 0.3 mg HCCA (IVD matrix HCCA-partitioned, Bruker Daltonics Bruker Daltonics) in 250 µL of Bruker standard solvent for MALDI (Sigma-Aldrich Chemie GmbH, Taufkirchen, Germany). Each sample was applied to eight different spots. Samples were allowed to dry for several minutes before MS measurements were taken using a Microflex LT instrument (Bruker Daltonics GmbH and Co. KG, Bremen, Germany). Measurements were taken in linear positive mode at a laser frequency of 60 Hz covering the molecular mass range of 2000 to 20,000 Da. The IS1 voltage was 19.96 kV, the IS2 voltage was maintained at 18.221 kV, and the lens voltage was 6 kV. Three single mass spectra from 500 laser shots each were generated per spot, thus 24 mass spectra per strain were produced. The instrument was calibrated using a standard calibrant mixture (Bruker Daltonics GmbH and Co. KG, Bremen, Germany), including *Escherichia coli* extracts, RNAase A, and myoglobin. The standard mixture was prepared according to the manufacturer’s recommendation and directly transferred to designated spots on the target slide.

### 2.6. Spectral Pre-Processing and Cleaning

Raw spectra were collected and imported in RStudio using the “MALDIquant” and “MALDIquantForeign” R-packages [15]. Spectral quality control, transformation, smoothing, baseline reduction, normalization, and peak detection were performed according to Gibb and Strimmer [15]. The three technical replicates resulting from each spot and acquired automatically by the Microflex LT instrument were averaged. The detected peaks or variables were explored for the presence of missing values and outliers across the eight biological replicates. The *aggr* function within the “VIM” R-package was used to rank the variables by decreasing number of missing values, while a distance of two standard deviations from the mean of the variable was used to identify outliers. Thereafter, the dataset was prepared for ML analysis by filtering out outliers and missing values, and by averaging, for each individual spectral, data originating from the corresponding biological replicates. Feature names represented by the exact *m*/*z* values were rounded to three digits after the comma, and the string “Peak” was added to them. Finally, a peak matrix with sample ID and 436 feature value intensities was created (File S1, Supplementary Material). For visual inspection, spectra from whole-cell extracts of three strains per each *Brucella* spp. used in this study, and highlighting the similarities among them, are reported in Figure S1, Supplementary Material.

### 2.7. Consensus Feature Selection Strategy and Statistics

To identify from among the 436 MS peaks the most relevant for species classification, different feature selection strategies based on variable importance were applied and compared. In particular, the ratio of between-groups to within-groups sum of squares (BSS/WSS) described by Dudoit et al. [21] was computed to rank features by their discriminant power among classes. Furthermore, recursive feature elimination implemented in the *varImp* function from the R-package “Caret” was applied in combination with different ML algorithms including Mulitnominal Logistic Regression (MNR), Random Forest (RF) and Neural Network (NN). The “nnet”, “rf” and “multinom” methods were selected within the *train* function from the R-package “Caret” to fit the different ML models, while the *trainControl* function was applied to create trained model objects with a 10-fold cross-validation method. For each strategy, the top 50 important features were selected and a Venn diagram was created by means of the R-package “Limma”, to display the overlapping features from the different feature selection strategies.

Lavine’s homogeneity test and Shapiro–Wilk normality test were performed to assess feature homogeneity and distribution. Since most of the variables did not meet Anova assumptions, a Kruskal–Wallis rank sum test was applied along with the Wilcoxon test to identify the features that differed significantly among species. A *p* value of less than 0.05 was considered significant.

### 2.8. Unsupervised Data Analysis: k-Means and Hierarchical Agglomerative Clustering

Unsupervised learning is a process of attempting to find hidden structures or relationships in complex data independently from labels. However, because label information was available in this study, unsupervised algorithms were constructed on the 16 consensus features, to explore the separability of the different *Brucella* spp.

Two commonly used clustering algorithms, namely *k*-means clustering and hierarchical agglomerative clustering (HAC), were investigated as follows.

The *preProcess* function from the R-package “Caret” was used to center and scale the numerical features (Z-score scale). For *k*-means clustering, the standard *kmeans* function from the R-package “stats” was employed. To make the results reproducible and stable, the *set.seed* function was used, and the number of random starting assignments (*nstart* = 25) was specified. *K*-means were computed for a range of cluster centers (*k*) between three and seven. To estimate the optimal number of clusters and the quality of the clustering technique for the different *k* values, the silhouette coefficient (SC), a value ranging between −1 and 1, was computed using the *silhouette* function from the R-package “cluster”. A high SC was regarded as an indicator of good clustering. To illustrate the results of the best *k*-means model in a two-dimensional plane, the function *fviz_cluster* from the R-package “factoextra” was used. Similarly, HAC models with three to seven clusters were applied to the *Brucella* dataset, and the SC value was used to identify the best number of clusters. Since the choice of distance metric and linkage can have a significant effect on the results of agglomerative clustering, different distance metrics (Euclidean and Manhattan distance) and different linkage functions (Ward linkage, average linkage, and complete or maximal linkage) were explored using *dist* and *hclust* R-functions. The best model was selected using the SC value and the overall accuracy, indicating the fraction of bacterial spectra that were correctly classified. Hierarchical clustering with bootstrapping was generated using the “pvclust” R-package [22], while the package “RColorBrewer” was employed to represent the data in a heatmap format.

### 2.9. Supervised Data Analysis: General Workflow

In contrast to unsupervised ML methods, supervised learning uses labeled cases to help predict outcomes. All supervised ML algorithms were trained or fitted on consensus features identified by the four feature selection methods described in the “consensus feature selection strategy” paragraph.

**Scheme 1:**
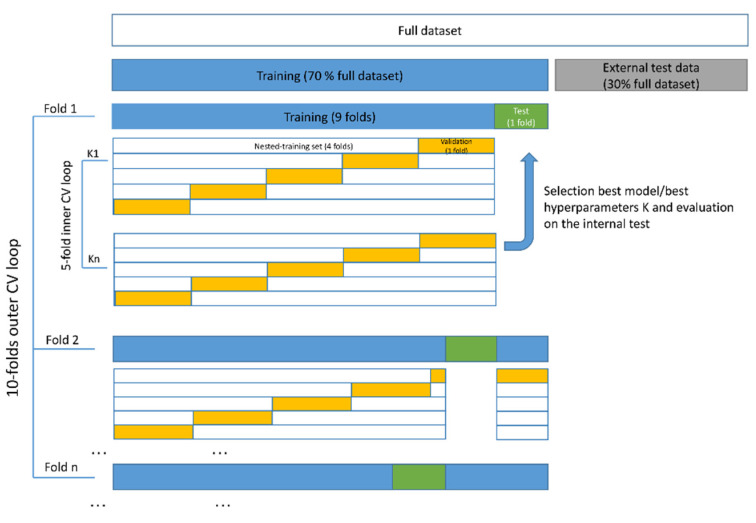
The MALDI-TOF MS dataset was randomly split in two independent training and external test datasets, representing the 70% and 30% of the cases, respectively. The external test dataset, encompassing 12 samples unseen during the modeling procedure and treated as unknown, was used to access the true performance of the developed model in a real-world setting. The training dataset was used to create fine-tuned ML models and to assess their expected performance by means of nested cross validation (nCV) with a 10-fold outer loop and 5-fold inner loop. In the outer loop, the training set was split into 10 folds or groups of approximately equal size, nine of which were assigned to the training set (in blue) and one to the test set (in green). In the inner loop, the training set was additionally split into five folds, four of which were assign to the nested training set and one to the validation set (in orange). The nCV process was as follows: The models were configured on the nested training data using a combination of all hyperparameters in the grid, and the accuracy on the validation dataset was assessed. Validation folds were rotated until all groups had contributed to the validation data. The best hyperparameters were identified as those with the highest validation performance over the five folds. The best hyperparameters were selected to train a classifier from the training dataset of the outer loop, and evaluate the expected performance of the model on the corresponding test dataset (green fold). The test fold was rotated among all folds of the outer CV loop to evaluate the ability of each model to generalize against new data.

To build an effective ML model from MALDI-TOF MS data, a general workflow based on the R-package “Caret” (classification and regression training) was employed. Randomly sampled cases were selected to create independent training and test datasets. Specifically, 70% of the cases were used as training dataset to create fine-tuned models and assess their expected performance. The remaining 30% were used as the external dataset, encompassing 12 samples unseen during the modeling procedure and treated as unknown, to evaluate the true performance of the model in a real-world setting. Before modeling, all numerical features in the whole dataset were centered and scaled (Z-score scale) using the *preProcess* function. Furthermore, to deal with class imbalance, up-sampling of the smaller cases was carried out using the *upSample* function, on the training dataset only. For each ML algorithm, a hyperparameter grid was defined based on the ML algorithm in the case at hand, while the metric chosen to select the optimal model for classification was “Accuracy”. Each fine-tuned ML model was trained using a nested cross validation (nCV) method with a 10-fold outer loop and 5-fold inner loop (Scheme 1). The inner CV loop was used to identify the best model hyperparameters for model selection, while the outer CV loop was to assess model generalization, describing the ability of the model to classify or forecast unseen data. To assess generalization, the mean of the metric across the folds of the outer CV loop and the corresponding standard deviation (STD) were computed, while rotating the training folds, to summarize the expected performance of the model. In general, a standard deviation of the mean of the accuracy with an order of magnitude smaller than the mean was used as indicator of a good generalizing model, and was thus suitable classifying unknown cases. To avoid an over-optimistic estimation of the model accuracy, two CV loops were performed using a different randomly sampled set of folds, which were defined using the *nested_cv* function from the R-package “rsample”. To reduce model complexity and avoid overfitting, a feature reduction step between the two CVs loops was performed. In particular, feature importance was evaluated using the *varImp* function, and new models were trained with variables showing the highest importance as determined by the ML model. At equal model performance, the simpler model with the better refined and reduced feature set was preferred to solve the *Brucella* classification problem.

The true performance of the final classifier was evaluated on the external test dataset (30% of the cases) (gray fold in Scheme 1) using the *predict* function. Based on these scores, a confusion matrix was computed, reporting the number of false positives (FP), false negatives (FN), true positives (TP), and true negatives (TN), together with the overall accuracy, a number between 0 and 1, indicating the fraction of bacterial spectra correctly classified.

### 2.10. Supervised ML Models: Neural Network, Random Forest, Support Vector Machine and Multinomial Logistic Regression

In this study four different supervised learning classifiers were used, namely Random Forest (RF), Neural Network (NN), Support Vector Machine (SVM), and Multinomial Logistic Regression (MNR). Accurate descriptions of the ML algorithms, and the relative advantages and disadvantages of the learning systems, are reported in Table S1 (Supplementary Material).

For each classifier, different models were trained according to the general workflow described in the dedicated paragraph (see above) with some model-specific differences. In particular, the *train* function with “*nnet*” and “*rf*” methods from the R-package “Caret” was employed to build the corresponding NN and RF models. To fit a fine-tuned NN model, the best hyperparameters “size” (=number of units in hidden layer) and “decay” (=regularization parameter to avoid overfitting) were searched among a grid of values ranging between 1 and 10 with a step of 1, and between 0 and 0.5 with a step of 0.1, respectively. For RF, the best “mtry” (=number of variables randomly sampled as candidates at each split) was searched among the values 1 to 10, while the optimal number of trees in the forest was investigated at 100, 500 and 1000 of trees to conserve computational resources.

An SVM model with a radial basis function kernel was built using the *train* function from R-package “Caret” and the “SVMRadial” method. The optimal “C” hyperparameter (=cost value) was searched using tuneGrid argument values ranging between 0.1 and 1 with a step of 0.1, while the function *sigest* from R-package “kernlab” was employed to estimate the kernel parameter sigma during each model fit. The *train* function from R-package “Caret” with the “multinom” method was used to build an MNR model with the tuning hyperparameter “decay” ranging between 0 and 1 with a step of 0.1.

### 2.11. Consensus Feature Selection and Effect on Supervised ML Model Accuracy

To address the role of the consensus feature selection strategy in the accuracy of the learning systems, all ML algorithms were trained and optimized on the top four classifier-specific variables, defined by means of the *varImp* function from the R-package “Caret”, and compared to the corresponding ML models fitted on the consensus features. The number of variables selected was defined by the number of features yielding the highest ML model accuracy using the consensus approach.

## 3. Results

### 3.1. Identification of 16 Consensus Features

**Figure 1:**
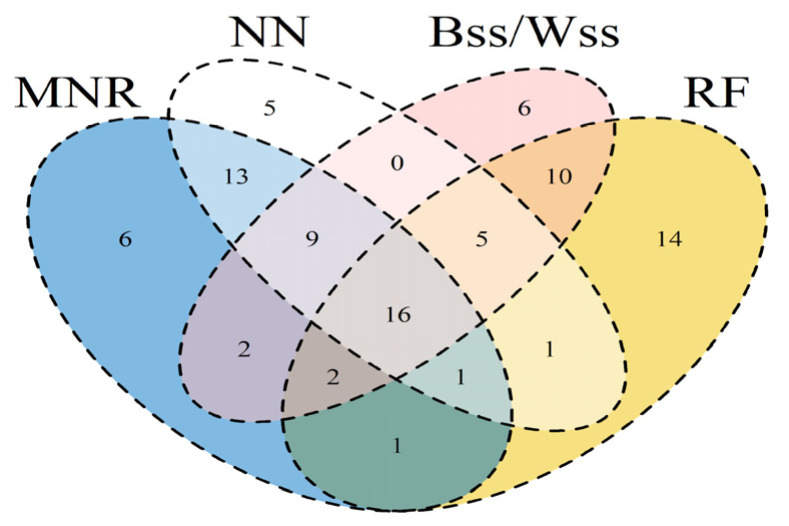
Venn diagram created using the R Limma package, displaying the level of overlap among different feature selection strategies based on variable importance assessed by means of Neural Network (NN), Multinomial Logistic Regression (MNR), Random Forest (RF) and the ratio of between-groups to within-groups sum of squares (BSS/WSS).

To identify from among the 436 variables reported in the final intensity matrix those most important for *Brucella* sp. classification, different feature selection methods based on variable importance were applied and compared. The graphical intersection of the results obtained with the different feature selection strategies is presented in Figure 1. In particular, the following 16 overlapping features were identified: Peak.6715, .2960, .9863, .13303, .8521, .9978, .7266, .9935, .7791, .5271, .3633, .4716, .4372, .8324, .8700, .4338. Details of unique and shared features among the different feature selection methods used are reported in Table S4, Supplementary Material.

Statistical analysis revealed that most of the consensus features differed significantly among the three *Brucella* spp., with *p*-values below 0.001 as shown in Table S5 (Supplementary Material), with exceptions for Peak.5271, .3633 and .2960. A graphical example of the distribution of Peak.6715 with corresponding Kruskal–Wallis testing and post hoc Wilcoxon testing is reported in Figure S2, Supplementary Material.

### 3.2. Three-Cluster Unsupervised ML Models Revealed a Good Brucella spp. Separation

**Figure 2:**
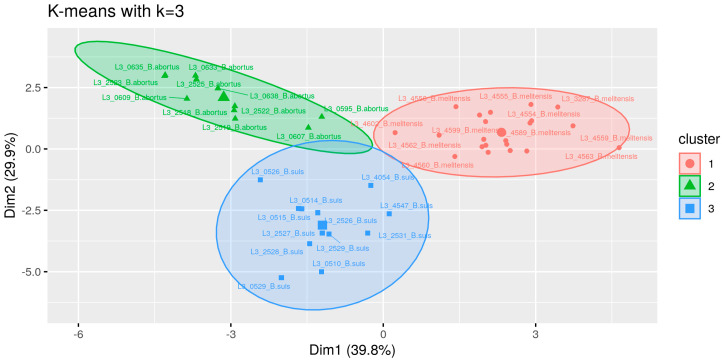
Illustration of the *k*-means model with the optimal number of clusters (*k* = 3). The function *fviz_cluster* from the R-package “factoextra” was used to plot the data points according to the first two principal components that explained the majority of the variance. The figure shows species-specific clustering. The optimal number of clusters is three, with an SC value of 0.39. The silhouette coefficient was computed using the *silhouette* function from the “cluster” R-package.

**Figure 3:**
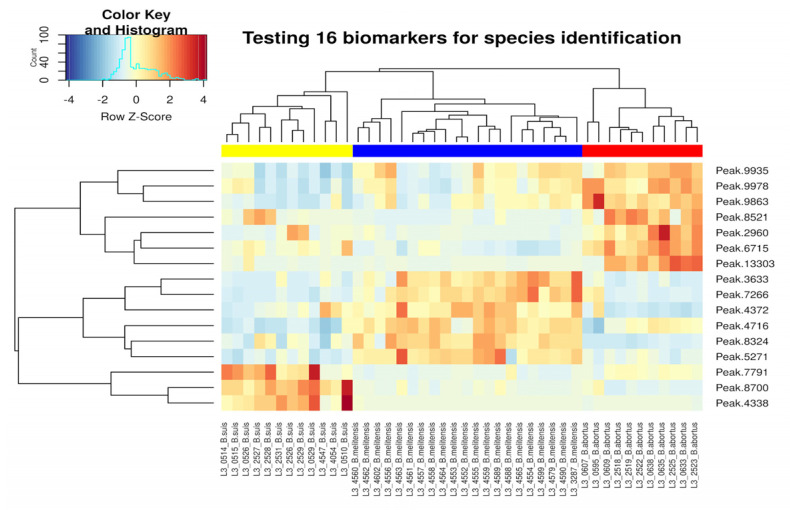
Heatmap displaying the intensities of the 16 potential biomarkers identified by means of a consensus feature selection method. Feature intensity was reported with the color code red for “high intensity peak” and blue for “low intensity peak”. Samples and features were ordered by means of agglomerative hierarchical clustering with average linkage and Manhattan distance.

Silhouette coefficients (SC) calculated over a range of five possible numbers of clusters (*k*) revealed the optimal *k*-means model for a *k* equal to three or four (Figure S3, Supplementary Material). Since *k* = 3 was concordant with the metadata, this model was preferred. The three-cluster *k*-means model exhibited a good division of cases, as shown in Figure 2. Similarly, the analysis of the SC values over a range of five possible numbers of clusters revealed the best HAC model was for a *k* value of three, with SC > 0.43 (Figure S4, Supplementary Material). Average linkage and Manhattan distance were identified as best metrics for class assignments of three-cluster agglomerative models, with an SC value of 0.48 (Figure S5, Supplementary Material). The final HAC model revealed clear species separation, as indicated by the corresponding high bootstrap values (Figure S6, Supplementary Material). Figure 3 presents the heatmap displaying the intensities of the 16 potential biomarkers used to feed the algorithm and the corresponding hierarchical clustering. The assignments produced by *k*-means and hierarchical clustering models with different cluster centers are reported in Tables S6 and S7, respectively (Supplementary Material).

### 3.3. Identification of the Optimal Fine-Tuned Supervised ML Models

All models built on the 16 consensus features displayed 100% overall accuracy for training CV folds and the external dataset (data not shown). The same results were achieved by reducing model complexity to four features for both SVM and NN, and to three features for RF and MNR, indicating that the corresponding 16-feature models were overfitted (Table 1). The most relevant predictors identified for *Brucella* spp. classification were Peak.3633, .8700, .6715, and .5271 for SVM; Peak.6715, .8324, .9863, and .8700 for NN; Peak.5271, .6715, .8700 ± .3633 for RF and Peak.9863, .6715, .8700 ± .9978 for MNR. The optimal hyperparameter values found for model tuning were *sigma* equal to 0.085 and *cost* equal to 0.1 for SVM, *size* equal to 2 and *decay* equal to 0.5 for NN, 1000 number of trees and *mtry* equal to 1 for RF, and *decay* equal to 1 for MNR. To view the performance of the different NN classifiers over the hyperparameter grid, see Figure S7, Supplementary Material.

The expected and true performance of the optimal fine-tuned models trained on the best three and four predictors are reported in Table 1. Independently from the final number of predictors, all ML models exhibited an expected accuracy of one, indicating that the model is likely to generalize well against new cases. The true performance of the final tuned model’s fit on the best four features revealed accurate prediction, with 100% overall accuracy for each *Brucella* species. In contrast, the accuracy of the fine-tuned model’s fit for only three features dropped down to 83 and 92%, for NN and SVM classifiers, respectively (Table 1). The confusion matrix showing the true performance of the fine-tuned NN model trained on the three most important variables is reported in Table S7, Supplementary Material.

### 3.4. Consensus Feature Selection Enhance the Supervised ML Model Accuracy

In comparison to the final models fitted and optimized on the consensus features, fine-tuned ML classifiers trained on the top four variables, defined by means of a ML-specific feature selection approach, revealed a reduction in performance as shown in Table 2. The analysis of the expected accuracy showed for these “alternative” ML models a generalization ability ranging between 0.95 for NN and 0.98 for SVM, while the true accuracy evaluated on the external test dataset was between 0.83 and 0.92, representing the incorrect classification of two strains or one, respectively, as shown in Table 3.

## 4. Discussion

In a recent contribution, Mesureur et al. showed that with a curated database (including 84 *Brucella* spp.), MALDI-TOF MS was able to identify *B. melitensis*, *B. abortus* and *B. suis* with an accuracy of 100%, 92.9%, and 100%, respectively [23]. In the present study, we showed how the use of supervised and unsupervised ML algorithms on the MALDI-TOF MS spectra of 44 *Brucella* spp. allowed refinement of the classification method, with an accuracy of 100% for each target species.

To create highly accurate ML models, the high dimensionality of the MS data, represented in the case at hand by 436 peaks (referred to in the text as features or explanatory variables), was reduced retaining only meaningful features. Different techniques are available for feature selection and include the use of filters, wrappers, or embedded methods with basic differences in how feature selection and classification tasks are accomplished [24,25,26]. One of the most straightforward methods for performing feature selection is based on the use of classifiers with built-in feature selection capability, e.g., models that can be accessed using the *train* function from the R-package “Caret”. A more intuitive approach, broadly used to find relevant variables and biomarkers for predicting disease (e.g., cancer datasets), ranks features based on the ability to discriminate between groups, using a univariate statistic such as between-group to within-group sum of squares (BSS/WSS) [26]. Here, a consensus feature selection strategy was applied, combining the BSS/WSS approach with the use of classifiers implementing the *varImp* function from the R-package “Caret”. Sixteen consensus features were identified as shown in the Venn diagram (Figure 1), displaying the number of features selected by each selection approach and the levels of overlap between them. The consensus approach allowed the selection of variables without resorting to stringent cut-off values, which can significantly affect the selected feature subset and therefore the performance of the learning system. Furthermore, the variables identified could be used for both supervised and unsupervised classifiers. Nevertheless, this systematic approach allowed the identification not only of features that yielded the best possible classifiers, but also of features whose expression may play a role in the differentiation of the three *Brucella* species. However, the proteins behind the 16 consensus features were not investigated, and therefore the biological interpretation of the most important features for species classification, including protein ID, functional annotations, or biological pathways (which might provide insight into the underlying biology of the species) remains an open issue.

Unsupervised methods such as *k*-means and hierarchical agglomerative clustering (HAC) are label-independent clustering techniques that are normally applied to find hidden structures in multivariable datasets. In our case, due to label availability, unsupervised learning systems were constructed on the 16 consensus features, to explore the separability of the different *Brucella* spp. Both unsupervised ML methods identified three optimal clustering numbers, with SC coefficients of 0.4 and 0.49 for *k*-means and HAC, respectively, demonstrating that hierarchical clustering allowed a finer division of cases. However, both models defined a clear split of the samples into the three *Brucella* species, as shown in Figure 2 and Figure 3. Interestingly, the heatmap in Figure 3 allows not only clear differentiation among the three target *Brucella* spp. but also identification within the same species of subgroups with different peak intensity, which may be linked to a different phenotypic profile (not investigated).

Previous studies showed that high-dimensional data with a small number of samples may lead to overoptimistic performance estimates for ML algorithms [27,28], especially if combined with simple validation methods like *k*-folds CV, which do not ensure a clear distinction between data used to validate the classifiers and data used for model development [29]. However, Vabalas and collaborators investigated different validation methods with different sample sizes, and demonstrated that a nested CV and train–test split approach produced robust and unbiased performance estimates regardless of sample size [16]. For this reason, to ensure a non-biased estimation of the classification accuracy of our ML models, both validation methods, nCV and train–test split, were implemented in our model development workflow. Indeed, the MALDI-TOF MS dataset was randomly split in two independent training and external test datasets, representing the 70% and 30% of the samples, respectively. The external test dataset, encompassing 12 samples unseen during the modeling procedure and treated as unknown, was used to access the true performance of the developed model in a real-world setting. The training dataset was used separately to create fine-tuned ML models and to assess their expected performance by nested cross validation (nCV) with a 10-fold outer loop and 5-fold inner loop. Notice that each fine-tuned ML model was further optimized to avoid overfitting, adding a feature reduction step between the two CV loops.

The final ML models’ fits on four features were able to correctly predict the species in the external unseen dataset, with 100% accuracy. No substantial performance differences were found among the best final NN, RF, SVM, and MNR classifiers, as shown in Table 1. However, the fits of RF and MNR models on three features overperformed the other classifiers with 100% true accuracy (Table 1). Not surprisingly, each ML classifier had different predictors since they were based on different algorithms. However, Peak.8700 and Peak.6715 were identified as important features by all four ML algorithms, suggesting a biological role in the species differentiation.

The excellent predictive performance achieved by our final classifiers is the consequence of the implementation of a good consensus feature selection strategy, which allowed an accurate and automated variable selection for ML modeling. Comparison of the accuracies of the learning systems fitted on features identified by consensus feature selection with those fitted by means of independent ML-specific feature selection, revealed in the latter case a reduction of both expected and true performance of the learning system. In particular, the overall accuracy of NN, RF, SVM, and MNR on unseen test dataset was reduced to 0.83 or 0.92, representing the incorrect classification of two strains or one, respectively (Table 2).

Although the consensus feature selection strategy played an important role in the accuracy of a learning system, the method itself did not prevent overfitting. This was addressed applying the feature reduction step between the two nCV loops during the fine-tune modeling process. Indeed, a model trained on only three or four features achieved the same accuracy as a more complex model with 16 features (Table 2). Thus, the use of a feature reduction step is highly recommended to increase the robustness of a supervised ML model.

## 5. Conclusions

In this work we have introduced an efficient and meaningful approach to classify unknown Brucella spp. from MALDI-TOF MS data, that goes beyond the methods suggested in earlier works. In particular, we showed how the implementation of a consensus feature selection strategy allowed the construction of robust supervised and unsupervised ML models, enabling highly accurate bacterial species recognition from MALDI-TOF MS data. In future, our whole procedure can be extended to other microbial species relevant in clinical settings.

## Figures and Tables

**Table 1:** Expected and true performance of the best final classifiers, optimized in terms of feature and model hyperparameters. The expected ML model accuracy was evaluated across all 10 CV folds of the training dataset, while the true ML accuracy was assessed on the external test dataset.

ML Model	Feature Number	Features	Best Model Hyperparam	Expected Accuracy	True Accuracy
SVM	4	Peak.3633, .8700, .6715, .5271	sigma = 0.57 cost = 0.1	1	1
	3	Peak.3633, .8700, .6715	sigma = 0.085 cost =0.1	1	0.92
NN	4	Peak.6715, .8324, .9863, .8700	size = 2 decay = 0.5	1	1
	3	Peak.6715, .8324, .9863	size = 3 decay = 0	1	0.83
RF	4	Peak.5271, .6715, .8700, .3633	ntree= 500 mtry = 1	1	1
	3	Peak.5271, .6715, .8700	ntree= 1000 mtry = 1	1	1
MNR	4	Peak.9863, .6715, .8700, .9978	decay = 1	1	1
	3	Peak.9863, .6715, .8700	decay = 1	1	1

**Table 2:** Performance of optimized fine-tuned ML models trained on the top four features identified using a feature selection approach specific for Mulitnomial Logistic Regression (MNR), Random Forest (RF), Neural Network (NN), and Support Vector Machine (SVM), separately. The performance was evaluated in terms of expected and true accuracy for training CV folds and external test datasets, respectively. The top four features used to train the corresponding ML model were also included.

	MNR	RF	NN	SVM
Features	Peak.3427, .4911, .9863, .4930	Peak.3427, .4351, .3677, .9978	Peak.4911, .6715, .4930, .8324	Peak.2880, .9863, .7377, .4249
Expected accuracy	0.98	0.95	0.96	0.96
True accuracy	0.833	0.833	0.92	0.833

**Table 3:** Species assignment for each strain from the external dataset, performed by the optimized fine-tuned ML models trained on the top four features identified using a standard feature selection approach specific for Mulitnomial Logistic Regression (MNR), Random Forest (RF), Neural Network (NN) and Support Vector Machine (SVM), separately. Incorrect classification is shown in bold.

		SVM		NN		RF		MNR	
Strain	Species	Score	Identity	Score	Identity	Score	Identity	Score	Identity
L3-0515	*B. suis*	*B. melitensis*	**incorrect**	*B. suis*	correct	*B. melitensis*	**incorrect**	*B. suis*	correct
L3-0529	*B. suis*	*B. suis*	correct	*B. suis*	correct	*B. suis*	correct	*B. suis*	correct
L3-2529	*B. suis*	*B. suis*	correct	*B. abortus*	**incorrect**	*B. suis*	correct	*B. suis*	correct
L3-0638	*B. abortus*	*B. melitensis*	**incorrect**	*B. abortus*	correct	*B. abortus*	correct	*B. abortus*	correct
L3-2519	*B. abortus*	*B. abortus*	correct	*B. abortus*	correct	*B. abortus*	correct	*B. abortus*	correct
L3-2525	*B. abortus*	*B. abortus*	correct	*B. abortus*	correct	*B. abortus*	correct	*B. abortus*	correct
L3-4556	*B. melitensis*	*B. melitensis*	correct	*B. melitensis*	correct	*B. melitensis*	correct	*B. melitensis*	correct
L3-4561	*B. melitensis*	*B. melitensis*	correct	*B. melitensis*	correct	*B. melitensis*	correct	*B.suis*	**incorrect**
L3-4564	*B. melitensis*	*B. melitensis*	correct	*B. melitensis*	correct	*B. melitensis*	correct	*B. suis*	**incorrect**
L3-4565	*B. melitensis*	*B. melitensis*	correct	*B. melitensis*	correct	*B. melitensis*	correct	*B. melitensis*	correct
L3-4590	*B. melitensis*	*B. melitensis*	correct	*B. melitensis*	correct	*B. melitensis*	correct	*B. melitensis*	correct
L3-4602	*B. melitensis*	*B. melitensis*	correct	*B. suis*	**incorrect**	*B. melitensis*	correct	*B. melitensis*	correct

## Data Availability

The data presented in this study are available on request from the corresponding author.

## Supplementary Materials

The following supporting information can be downloaded at: https://www.mdpi.com/article/10.3390/microorganisms10081658/s1, File S1: Intensity matrix “featureMatrix.csv” displaying 436 features for the whole *Brucella* dataset; Figure S1: MALDI-TOF MS spectra of whole-cell extracts of *B. melitensis*, *B. suis*, and *B. abortus*. Three strains for each species are shown; Figure S2: Box-plot displaying Peak.6715 for the Kruskal-Wallis and Wilcoxon tests; Figure S3: Average silhouette coefficient vs. *k*-means models. Silhouette coefficient (SC) over a range of possible number of clusters (k). Here, since SC is maximized for *k* = 3, this *k*-means model was preferred. The optimal number of clusters is three; Figure S4: Average silhouette coefficient vs. HAC model. Silhouette coefficient (SC) over a range of possible number of clusters (*k*). SC is maximized for *k* = 3 and *k* = 4. Since *k* = 3 is concordant with the metadata, this HAC model was preferred; Figure S5: Average silhouette coefficient vs. different distance–linkage combinations; Figure S6: Hierarchical agglomerative clustering tree based on MALDI-TOF MS data (16 features). Percentages of bootstrapping replicates supporting the location of individual nodes are indicated; Figure S7: The graph illustrates the performance of the different NN classifiers over the hyperparameter grid. (a) NN classifiers’ fit on the top four most important features, displaying best performance with size = 2 and decay = 0.5. (b) NN classifiers modeled on the top three most important features, showing best performance with size = 3 and decay = 0. Notice that the classifiers built on only three features are more sensitive to model parameter tuning; Table S1: Machine learning algorithms; Table S2: *Brucella* strains and isolates employed in this study; Table S3: List of all R-packages used in this work; Table S4: Intersection among the results of different feature selection strategies based on variable importance assessed by means of MNR, RF, NN, and BSS/WSS methods; Table S5: Levene’s homogeneity of variance, Shapiro–Wilk normality test on 16 consensus features, performed to verify that the Anova assumptions were met. Kruskal–Wallis and Wilcoxon tests were carried out to identify significant differences among species. Signif. codes: 0 ‘***’ 0.001 ‘**’ 0.01 ‘*’ 0.05 ‘.’ 0.1 ‘ ’ 1; Table S6: *k*-means clustering assignments; Table S7: Hierarchical agglomerative clustering assignments; Table S8: Confusion matrix showing the true performance of the fine-tuned NN model when applied to the external dataset encompassing 12 *Brucella* sp. The model was trained on 41 samples using the three most important features, namely Peak.6715, .8324, and .9863. The diagonal elements of the confusion matrix indicate correct predictions, while the off-diagonals represent incorrect predictions. The classifier was modeled with tune parameters size = 3 and decay = 0. The samples L3-2519 *B. abortus* and L3-4602 *B. melitensis* were both incorrectly classified as *B. suis*.
