# Supplementary material for: Machine Learning Algorithms for Classification of MALDI-TOF MS Spectra from Phylogenetically Closely Related Species Brucella melitensis, Brucella abortus and Brucella suis
Source: Microorganisms. 2022 Aug 17;10(8):1658. doi: 10.3390/microorganisms10081658 (PMC9416640; doi:10.3390/microorganisms10081658)

Figure S1: MALDI-TOF MS spectra of whole-cell extracts of *B. melitensis*, *B. suis*, and *B. abortus*. Three strains for each species are shown.

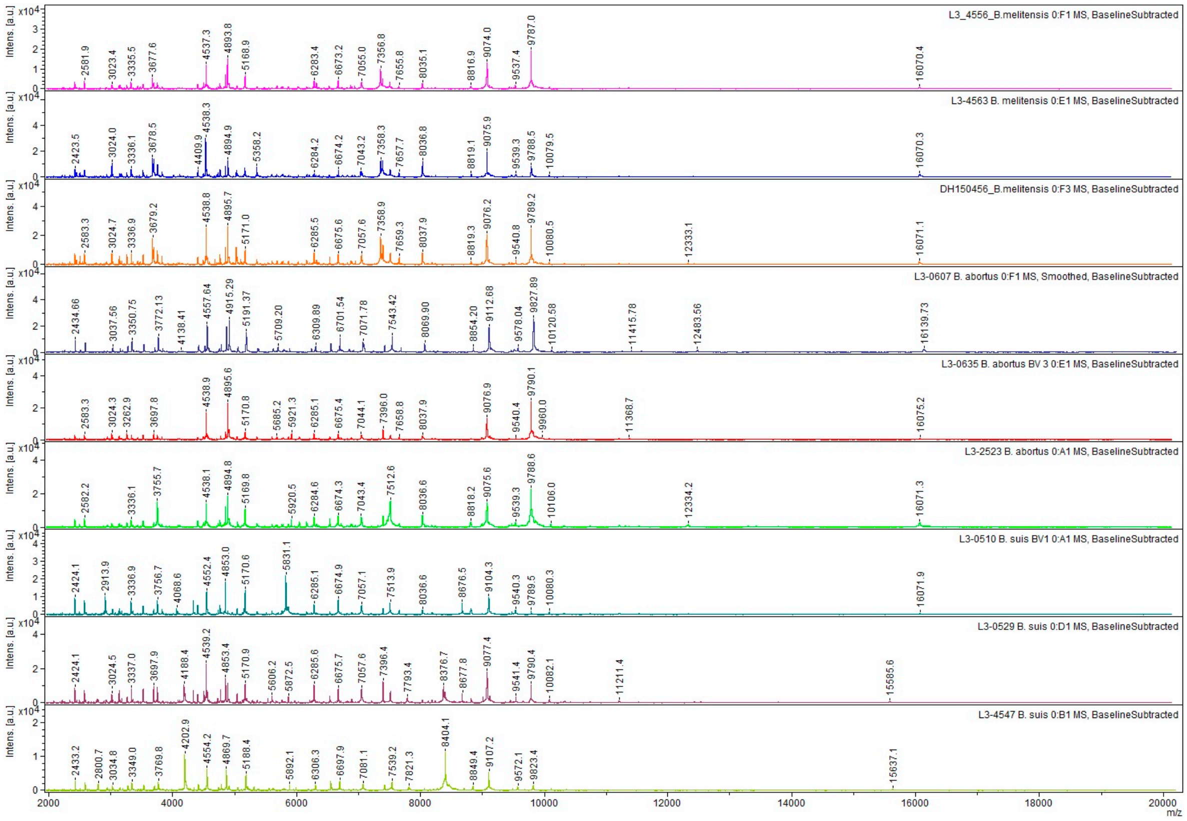

Supplement: Supplementary file 1 [file microorganisms-10-01658-s001.zip › Figure S1.pdf]
