# Supplementary material for: Machine Learning Algorithms for Classification of MALDI-TOF MS Spectra from Phylogenetically Closely Related Species Brucella melitensis, Brucella abortus and Brucella suis
Source: Microorganisms. 2022 Aug 17;10(8):1658. doi: 10.3390/microorganisms10081658 (PMC9416640; doi:10.3390/microorganisms10081658)

Figure S3: Average silhouette coefficient vs. k-means models. Silhouette coefficient (SC) over a range of possible number of clusters (k). Here, since SC is maximized for k = 3, this k-means model was preferred. The optimal number of clusters is three.

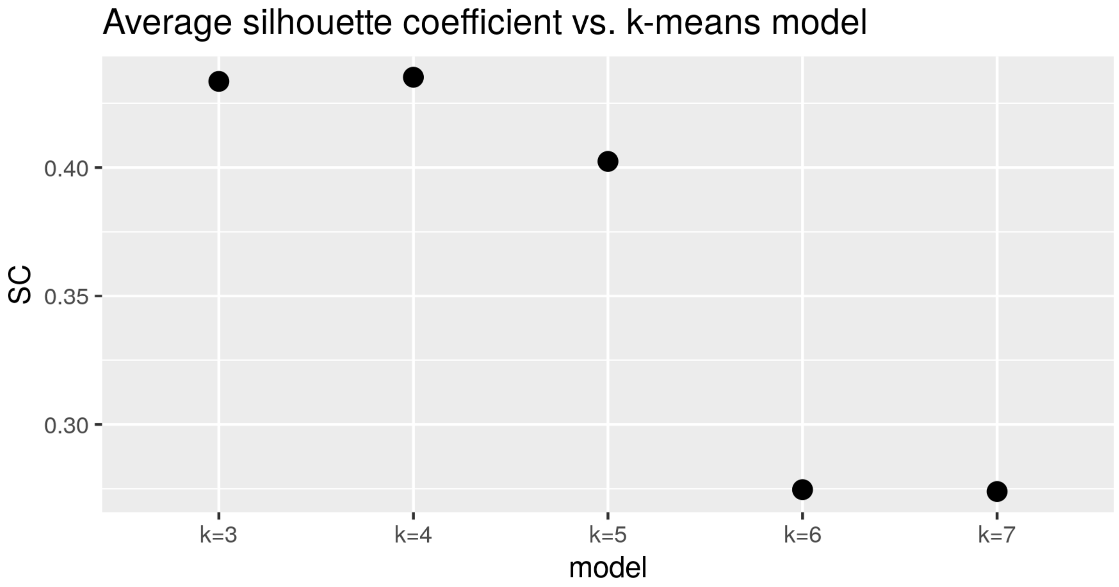

Supplement: Supplementary file 1 [file microorganisms-10-01658-s001.zip › Figure S3.pdf]
