# Supplementary material for: Machine Learning Algorithms for Classification of MALDI-TOF MS Spectra from Phylogenetically Closely Related Species Brucella melitensis, Brucella abortus and Brucella suis
Source: Microorganisms. 2022 Aug 17;10(8):1658. doi: 10.3390/microorganisms10081658 (PMC9416640; doi:10.3390/microorganisms10081658)

Figure S4: Average silhouette coefficient vs. HAC model. Silhouette coefficient (SC) over a range of possible number of clusters (k). SC is maximized for $k = 3$ and $k = 4$. Since $k = 3$ is concordant with the metadata, this HAC model was preferred.

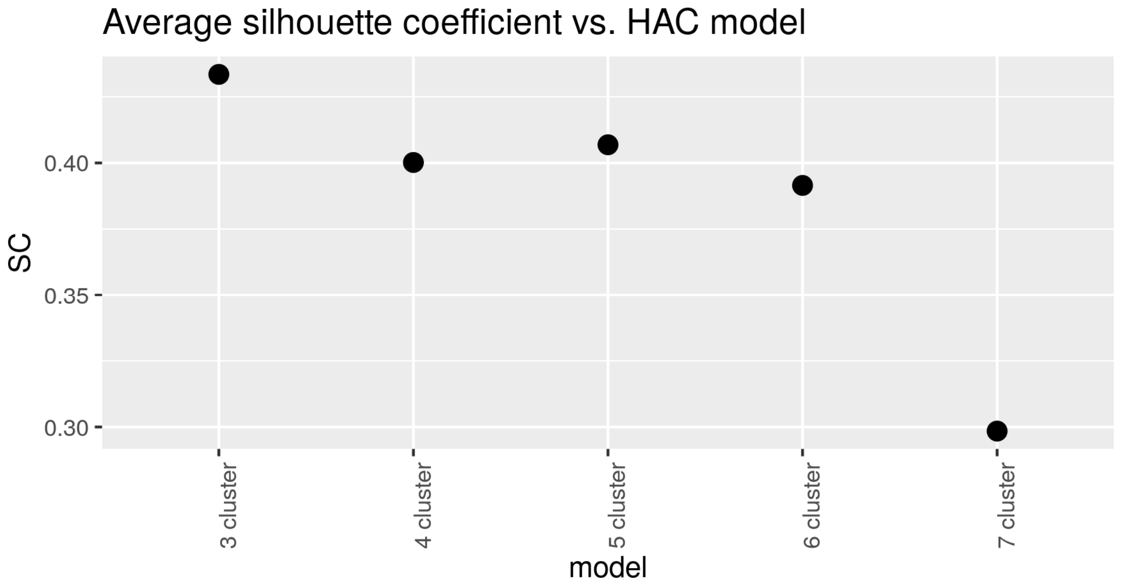

Supplement: Supplementary file 1 [file microorganisms-10-01658-s001.zip › Figure S4.pdf]
