# Supplementary material for: Machine Learning Algorithms for Classification of MALDI-TOF MS Spectra from Phylogenetically Closely Related Species Brucella melitensis, Brucella abortus and Brucella suis
Source: Microorganisms. 2022 Aug 17;10(8):1658. doi: 10.3390/microorganisms10081658 (PMC9416640; doi:10.3390/microorganisms10081658)

Figure S6: Hierarchical agglomerative clustering tree based on MALDI-TOF MS data (16 features). Percentages of bootstrapping replicates supporting the location of individual nodes are indicated.

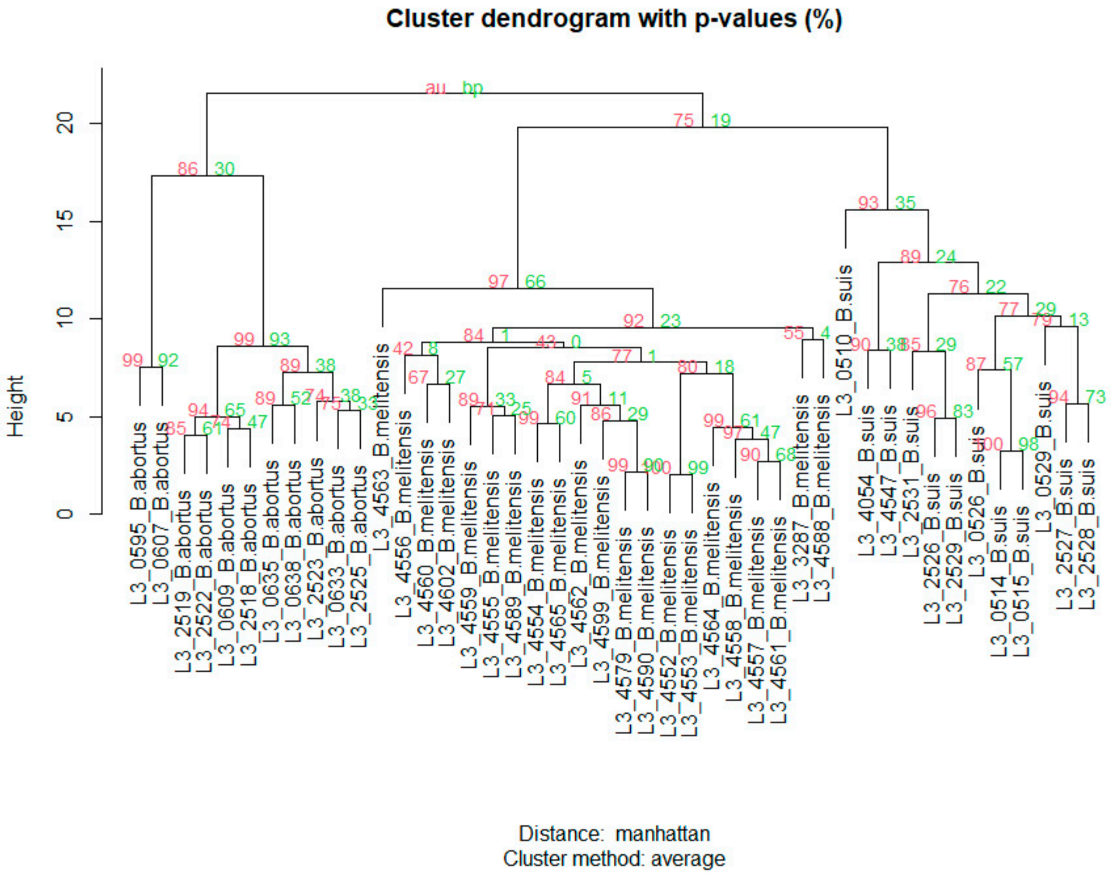

Supplement: Supplementary file 1 [file microorganisms-10-01658-s001.zip › Figure S6.pdf]
