# Supplementary material for: Machine Learning Algorithms for Classification of MALDI-TOF MS Spectra from Phylogenetically Closely Related Species Brucella melitensis, Brucella abortus and Brucella suis
Source: Microorganisms. 2022 Aug 17;10(8):1658. doi: 10.3390/microorganisms10081658 (PMC9416640; doi:10.3390/microorganisms10081658)

Figure S7: The graph illustrates the performance of the different NN classifiers over the hyperparameter grid. (a) NN classifiers' fit on the top four most important features, displaying best performance with size = 2 and decay = 0.5. (b) NN classifiers modeled on the top three most important features, showing best performance with size = 3 and decay = 0.

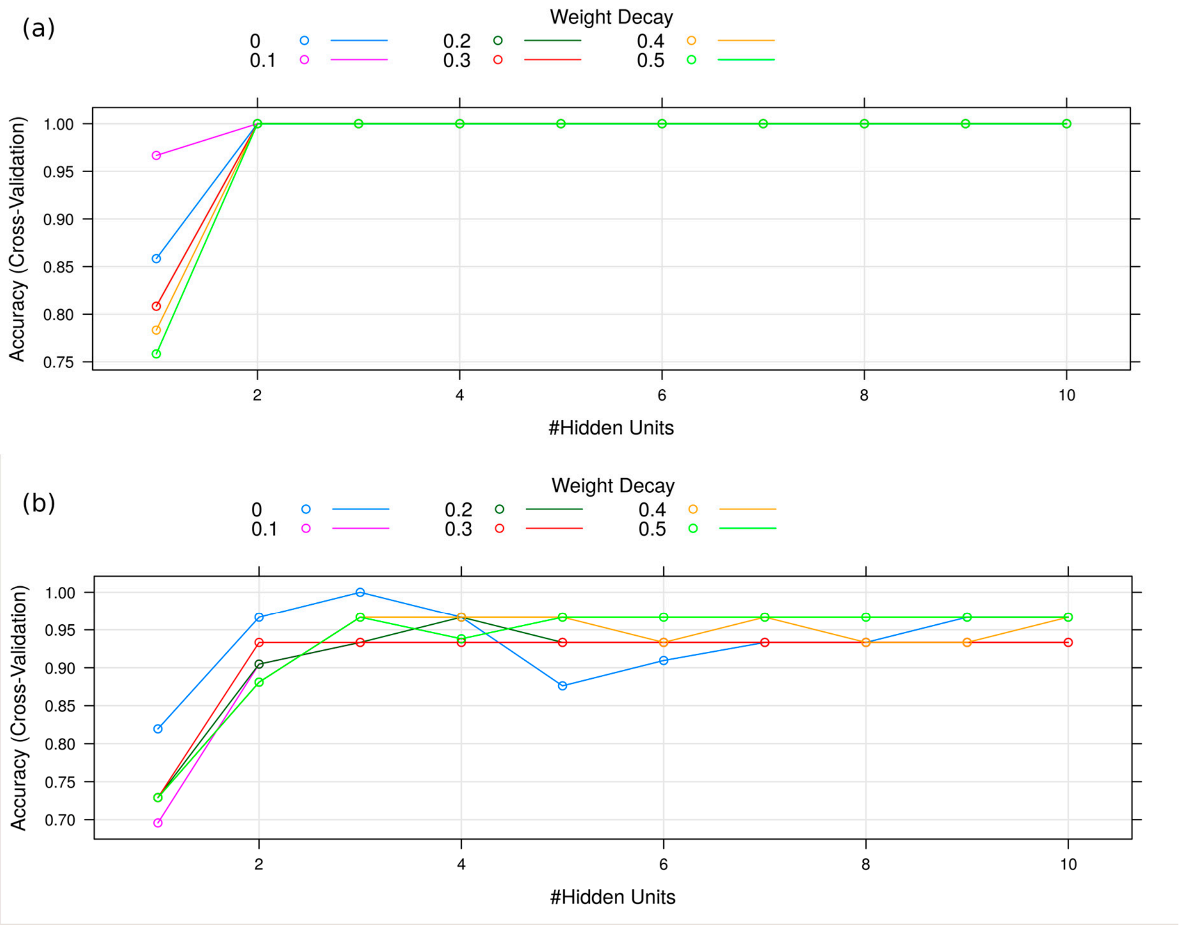

Supplement: Supplementary file 1 [file microorganisms-10-01658-s001.zip › Figure S7.pdf]
