# Supplementary material for: Machine Learning Algorithms for Classification of MALDI-TOF MS Spectra from Phylogenetically Closely Related Species Brucella melitensis, Brucella abortus and Brucella suis
Source: Microorganisms. 2022 Aug 17;10(8):1658. doi: 10.3390/microorganisms10081658 (PMC9416640; doi:10.3390/microorganisms10081658)
Supplement: Supplementary file 1 [file microorganisms-10-01658-s001.zip › Supplemental Tables.pdf]

Table S1. Machine learning algorithms

| | Algorithm | Description | Advantage | Drawbacks/Pitfalls/Weakness | Hyperparameters |
|---|---|---|---|---|---|
| **Unsupervised ML algorithms** | k-means | *k*-means method separate a dataset into k clusters such that the sum of squares from points to the assigned cluster centres is minimized. | 1. The method is massively scalable (relies only on basic linear algebra operations) and fast<br><br>2. it is very simple | 1. Assumes equal variance of the clusters, a fairly restrictive assumption. In practice, this criteria is almost never true<br><br>2. The k-means clustering requires the users to specify the number of clusters to be generated<br><br>3. The final results obtained are sensitive to the initial random selection of cluster centers.<br><br>4. Sensitive to outliers | - |
| | Agglomerative Hierarchical Clustering (AHC) | AHC creates clusters by a "bottom up" approach starting from singeltone clusters and merging, based on their similarity, the closest together to create bigger clusters. | 1. No assumption of a particular number of clusters (e.g. k-means)<br><br>2. Highly flexible in terms of type of data | 1. Distance metric (Euclidean or Manhattan) and linkage criteria (Ward, Average or Complete linkage) need to be specified<br><br>2. Not highly scalable when compared to K-means methods. | - |
| **Unsupervised ML algorithms** | Multinomial logistic regression (NMR) | It is an extention of binomial logistic regression that allow for a dependent variable with more than two classes. | 1. Does not assume a normal distribution of the variables | Assumption of independence among the dependent variable (no multicollinearity). | |
| | Support vector machine (SVM) using radial basis kernel | SVM attempts to maximally separate classes by finding the support vector with the lowest error rate or maximum separation. SVMs can use many types of kernel functions. The most common kernel functions are linear and the radial basis function or RBF. The linear basis function attempts to separate classes by finding hyperplanes in the feature space that maximally separate classes. The RBF uses set of local Gaussian shaped basis kernels to find a nonlinear separation of the classes.<br><br>An SVM works by projecting the data into a higher dimensional space and separating it into different classes by using a single (or set of) hyperplanes. | 1. anyone who runs it will theoretically get the same answer (that is not true for NN);<br><br>2. Can easily handle imbalanced data by re-weighting the points | 1. Slow<br><br>2. Do not scale huge dataset<br><br>3. Multicollinear variables need to be removed | - Sigma<br>- C |
| | Neural networks (NN) | NN uses one or more “hidden layers” of multiple “hidden units” to perform “function approximation”. | 1. high “model capacity”: the use of multiple hidden units in one or more layers, allows neural networks to | 1. Because of the large number of hidden units, neural networks have many “weights” or “parameters”, which often leads to overfitting of neural network models, which limits | - Size<br>- Decay |

| | | | | | |
|---|---|---|---|---|---|
| | | | approximate complex functions. | generalization. for good performance.<br><br>2. Computational complexity. | |
| | Random forest (RF) | RF is an ensamble learning method that works by generating a large number of individual decision trees. Each individual tree spits out a class prediction. The class with the most votes in the forest is the predicted class by the model. | 1. Random forest method is highly scalable and generally produces good results, even for complex problems.<br><br>2. Robust to noise or outliers in the training data. | | - mtry<br>- number or trees in the forest |

Table S2: *Brucella* strains and isolates employed in this study

| ID | Year of isolation | Origin | BCSP31 PCR / IS711 PCR | Bruce-ladder PCR and species designation |
|---|---|---|---|---|
| L3-0510 | | Type strain NCTC 103161330 | + / + | *B. suis* |
| L3-0514 | 1998 | unknown | + / + | *B. suis* |
| L3-0515 | 1998 | guinea pig | + / + | *B. suis* |
| L3-0526 | 2000 | wild rabbit | + / + | *B. suis* |
| L3-0529 | 1998 | wild boar | + / + | *B. suis* |
| L3-2526 | 2005 | unknown | + / + | *B. suis* |
| L3-2527 | 2005 | unknown | + / + | *B. suis* |
| L3-2528 | 2005 | unknown | + / + | *B. suis* |
| L3-2529 | 2005 | unknown | + / + | *B. suis* |
| L3-2531 | | Type strain NCTC 11996513 | + / + | *B. suis* |
| L3-4054 | | | + / + | *B. suis* |
| L3-4547 | 2014 | human | + / + | *B. suis* |
| L3-0595 | 1998 | unknown | + / + | *B. abortus* |
| L3-0607 | 2000 | unknown | + / + | *B. abortus* |
| L3-0609 | 1996 | cattle | + / + | *B. abortus* |
| L3-0633 | 1998 | human | + / + | *B. abortus* |
| L3-0635 | 1998 | cattle | + / + | *B. abortus* |
| L3-0638 | | type strain NCTC 10093544 | + / + | *B. abortus* |
| L3-2518 | 2005 | unknown | + / + | *B. abortus* |
| L3-2519 | 2005 | unknown | + / + | *B. abortus* |
| L3-2522 | 2005 | unknown | + / + | *B. abortus* |
| L3-2523 | 2005 | unknown | + / + | *B. abortus* |
| L3-2525 | 2005 | unknown | + / + | *B. abortus* |
| L3-3287 | 2010 | human | + / + | *B. melitensis* |
| L3-4552 | 2014 | human | + / + | *B. melitensis* |
| L3-4553 | 2014 | human | + / + | *B. melitensis* |
| L3-4554 | 2014 | human | + / + | *B. melitensis* |
| L3-4555 | 2014 | human | + / + | *B. melitensis* |
| L3-4556 | 2014 | human | + / + | *B. melitensis* |
| L3-4557 | 2014 | human | + / + | *B. melitensis* |
| L3-4558 | 2014 | human | + / + | *B. melitensis* |
| L3-4559 | 2014 | human | + / + | *B. melitensis* |
| L3-4560 | 2014 | human | + / + | *B. melitensis* |
| L3-4561 | 2014 | human | + / + | *B. melitensis* |
| L3-4562 | 2014 | human | + / + | *B. melitensis* |
| L3-4563 | 2014 | human | + / + | *B. melitensis* |
| L3-4564 | 2014 | human | + / + | *B. melitensis* |
| L3-4565 | 2014 | human | + / + | *B. melitensis* |
| L3-4579 | 2015 | human | + / + | *B. melitensis* |
| L3-4588 | 2015 | human | + / + | *B. melitensis* |
| L3-4589 | 2015 | human | + / + | *B. melitensis* |
| L3-4590 | 2015 | human | + / + | *B. melitensis* |
| L3-4599 | 2015 | human | + / + | *B. melitensis* |
| L3-4602 | 2015 | human | + / + | *B. melitensis* |

Table S3: List of all R-packages used in this work

| R-packages | Source |
|---|---|
| MALDIquant | https://github.com/sgibb/MALDIquant/ |
| MALDIquantForeign | https://github.com/sgibb/MALDIquantForeign/ |
| Caret | https://github.com/topepo/caret/ |
| Limma | https://github.com/gangwug/limm |
| rsample | https://github.com/cran/rsample |
| kernlab | https://github.com/cran/kernlab |
| MLmetrics | https://github.com/cran/MLmetrics/blob/master/R/MLmetrics.R |
| VennDiagram | https://github.com/cran/VennDiagram |
| gridExtra | https://github.com/cran/gridExtra |
| repr | https://github.com/cran/repr |
| dylyr | https://github.com/tidyverse/dplyr |
| tidyverse | https://github.com/tidyverse |
| ggplot2 | https://github.com/cran/ggplot2 |
| cluster | https://github.com/cran/cluster |
| factoextra | https://github.com/cran/factoextra |
| pvclust | https://github.com/cran/pvclust |
| RcoloBrewer | https://github.com/cran/RColorBrewer |
| GGally | https://github.com/ggobi/ggally |
| stats | https://github.com/topics/r-stats |
| ggpubr | https://github.com/cran/ggpubr |

Table S4. Intersection among the results of different feature selection strategies based on variable importance assessed by means of MNR, RF, NN and Bss/Wss.

| **Methods** | **Features** |
|---|---|
| MNR:RF:NN:BssWss | "Peak.6715" "Peak.2960" "Peak.9863" "Peak.13303" "Peak.8521" "Peak.9978" "Peak.7266" "Peak.9935" "Peak.7791" "Peak.5271" "Peak.3633" "Peak.4716" "Peak.4372" "Peak.8324" "Peak.8700" "Peak.4338" |
| RF:NN:BssWss | "Peak.2327" "Peak.8682" "Peak.3897" "Peak.9813" "Peak.9633" |
| MNR:RF:BssWss | "Peak.3427" "Peak.10240" |
| MNR:NN:BssWss | "Peak.13434" "Peak.19580" "Peak.5922" "Peak.7576" "Peak.3605" "Peak.7472" "Peak.6050" "Peak.9431" "Peak.6657" |
| MNR:RF:NN | "Peak.3234" |
| MNR:BssWss | "Peak.6367" "Peak.7216" |
| RF:NN | "Peak.3930" |
| MNR:NN | "Peak.5956" "Peak.4161" "Peak.3989" "Peak.8654" "Peak.18867" "Peak.9034" "Peak.4911" "Peak.4930" "Peak.7455" "Peak.8298" "Peak.7008" "Peak.12930" "Peak.5913" |
| MNR:RF | "Peak.11843" |
| RF:BssWss | "Peak.3677" "Peak.7355" "Peak.2551" "Peak.4351" "Peak.4249" "Peak.9647" "Peak.3474" "Peak.7377" "Peak.2121" "Peak.3313" |
| MNR | "Peak.7160" "Peak.13488" "Peak.4948" "Peak.9746" "Peak.3788" "Peak.6550" |
| RF | "Peak.2880" "Peak.4006" "Peak.19298" "Peak.8015" "Peak.5197" "Peak.9889" "Peak.4432" "Peak.5545" "Peak.3737" "Peak.5817" "Peak.4313" "Peak.7890" "Peak.4189" "Peak.8549" |
| NN | "Peak.5657" "Peak.2166" "Peak.2488" "Peak.1999" "Peak.11311" |
| BssWss | "Peak.2451" "Peak.3016" "Peak.19267" "Peak.4810" "Peak.2842" "Peak.3829" |

Table S5: Levene´ s homogeneity of variance, Shapiro-Wilk normality test on 16 consensus features were performed to verify that the Anova assumtion were met. Kruskal-Wallis and the Wilcoxon tests were used to identify significant differences among species. Signif. Codes: 0 '***' 0.001 '**' 0.01 '*' 0.05 '.' 0.1 ' ' 1

| **Feature** | **Levene´s homogeneity test** | **Shapiro-Wilk normality test on residuals (p-value)** | **Krustal-Wallis test (p-value)** | **Wilcoxon test** | | |
|---|---|---|---|---|---|---|
| | | | | *B.mel-B.abo* | *B.suis-B.abo* | *B.suis-B.mel* |
| Peak.6715 | 0.07896 | 0.2663 | 1.4e-07 *** | 1.6e-08 | 4.4e-07 | 3.8e-05 |
| Peak.2960 | 0.002237 ** | 2.048e-05 | 4.777e-05 *** | 3.1e-08 | 0.0022 | **0.62** |
| Peak.9863 | 0.4508 | 0.007161 | 8.621e-07 *** | 3e-06 | 1.5e-06 | 0.00069 |
| Peak.13303 | 1.61e-05 *** | 1.87e-08 | 0.0008986 *** | 0.0027 | 0.0086 | 0.0054 |
| Peak.8521 | 0.002446 ** | 0.005418 | 6.491e-06 *** | 3.9e-05 | 0.044 | 8.7e-06 |
| Peak.9978 | 0.7531 | 0.7531 | 2.3e-05 **** | 0.00019 | 1.8e-05 | 0.0078 |
| Peak.7266 | 0.03454 * | 0.002468 | 5.552e-07 *** | 7.8e-06 | 5.6e-07 | 2.3e-08 |
| Peak.9935 | 0.4861 | 0.7244 | 1.8e-05 *** | 0.0095 | 1e-05 | 0.00016 |
| Peak.7791 | 2.558e-08 *** | 2.988e-05 | 2.55e-06 *** | 0.00051 | 0.011 | 2.5e-07 |
| Peak.5271 | 0.1104 | 0.000139 | 1.1e-06 *** | 3e-06 | **0.051** | 1.5e-06 |
| Peak.3633 | 0.02254 * | 0.01517 | 6.945e-07 *** | 1e-06 | **0.79** | 2.5e-07 |
| Peak.4716 | 0.6745 | 0.1029 | 1.2e-06 *** | 0.00035 | 0.013 | 3.9e-08 |
| Peak.4372 | 0.2596 | 0.0003244 | 4.92e-06 *** | 5.7e-06 | 0.00098 | 0.00041 |
| Peak.8324 | 0.007214 ** | 0.6868 | 4.1e-07 *** | 1.6e-08 | 4.1e-07 | 5.1e-06 |
| Peak.8700 | 0.004715 ** | 5.441e-06 | 4.567e-07 *** | 0.0056 | 3e-06 | 5.6e-09 |
| Peak.4338 | 1.245e-06 *** | 2.832e-09 | 3.204e-06 *** | 0.00012 | 0.00014 | 1.9e-05 |

Table S6: K-means assignments

| ID | Species | k3_assign. | k4_assign. | k5_assign. | k6_assign. | k7_assign. |
|---|---|---|---|---|---|---|
| L3-0510 | *B. suis* | 3 | 4 | 4 | 4 | 7 |
| L3-0514 | *B. suis* | 3 | 4 | 4 | 1 | 6 |
| L3-0515 | *B. suis* | 3 | 4 | 4 | 1 | 6 |
| L3-0526 | *B. suis* | 3 | 4 | 4 | 1 | 6 |
| L3-0529 | *B. suis* | 3 | 4 | 4 | 1 | 6 |
| L3-0595 | *B. abortus* | 2 | 3 | 3 | 3 | 4 |
| L3-0607 | *B. abortus* | 2 | 3 | 3 | 3 | 4 |
| L3-0609 | *B. abortus* | 2 | 2 | 2 | 6 | 2 |
| L3-0633 | *B. abortus* | 2 | 2 | 2 | 6 | 2 |
| L3-0635 | *B. abortus* | 2 | 2 | 2 | 6 | 2 |
| L3-0638 | *B. abortus* | 2 | 2 | 2 | 6 | 2 |
| L3-2518 | *B. abortus* | 2 | 2 | 2 | 6 | 2 |
| L3-2519 | *B. abortus* | 2 | 2 | 2 | 6 | 2 |
| L3-2522 | *B. abortus* | 2 | 2 | 2 | 6 | 2 |
| L3-2523 | *B. abortus* | 2 | 2 | 2 | 6 | 2 |
| L3-2525 | *B. abortus* | 2 | 2 | 2 | 6 | 2 |
| L3-2526 | *B. suis* | 3 | 4 | 4 | 4 | 7 |
| L3-2527 | *B. suis* | 3 | 4 | 4 | 1 | 6 |
| L3-2528 | *B. suis* | 3 | 4 | 4 | 1 | 6 |
| L3-2529 | *B. suis* | 3 | 4 | 4 | 4 | 7 |
| L3-2531 | *B. suis* | 3 | 4 | 4 | 4 | 7 |
| L3-3287 | *B. melitensis* | 1 | 1 | 1 | 5 | 5 |
| L3-4054 | *B. suis* | 3 | 3 | 3 | 3 | 3 |
| L3-4547 | *B. suis* | 3 | 4 | 4 | 1 | 3 |
| L3-4552 | *B. melitensis* | 1 | 1 | 1 | 5 | 5 |
| L3-4553 | *B. melitensis* | 1 | 1 | 1 | 5 | 5 |
| L3-4554 | *B. melitensis* | 1 | 1 | 1 | 5 | 5 |

| | | | | | | |
|---|---|---|---|---|---|---|
| L3-4556 | *B. melitensis* | 1 | 1 | 1 | 5 | 5 |
| L3-4557 | *B. melitensis* | 1 | 1 | 1 | 5 | 5 |
| L3-4558 | *B. melitensis* | 1 | 1 | 5 | 2 | 1 |
| L3-4559 | *B. melitensis* | 1 | 1 | 5 | 2 | 1 |
| L3-4560 | *B. melitensis* | 1 | 1 | 1 | 5 | 5 |
| L3-4561 | *B. melitensis* | 1 | 1 | 5 | 2 | 1 |
| L3-4562 | *B. melitensis* | 1 | 1 | 1 | 5 | 5 |
| L3-4563 | *B. melitensis* | 1 | 1 | 5 | 2 | 1 |
| L3-4564 | *B. melitensis* | 1 | 1 | 5 | 2 | 1 |
| L3-4565 | *B. melitensis* | 1 | 1 | 1 | 5 | 5 |
| L3-4579 | *B. melitensis* | 1 | 1 | 1 | 5 | 5 |
| L3-4588 | *B. melitensis* | 1 | 1 | 1 | 5 | 5 |
| L3-4589 | *B. melitensis* | 1 | 1 | 1 | 5 | 5 |
| L3-4590 | *B. melitensis* | 1 | 1 | 1 | 5 | 5 |
| L3-4599 | *B. melitensis* | 1 | 1 | 1 | 5 | 5 |
| L3-4602 | *B. melitensis* | 1 | 1 | 1 | 5 | 5 |

Table S7: Hierarchical agglomerative clustering assignments

| ID | Species | agg3_assign. | agg4_assign. | agg5_assign. | agg6_assign. | agg7_assign. |
|---|---|---|---|---|---|---|
| L3-0510 | *B. suis* | 1 | 1 | 1 | 1 | 1 |
| L3-0514 | *B. suis* | 1 | 1 | 1 | 1 | 2 |
| L3-0515 | *B. suis* | 1 | 1 | 1 | 1 | 2 |
| L3-0526 | *B. suis* | 1 | 1 | 1 | 1 | 2 |
| L3-0529 | *B. suis* | 1 | 1 | 1 | 1 | 2 |
| L3-0595 | *B. abortus* | 2 | 2 | 2 | 2 | 3 |
| L3-0607 | *B. abortus* | 2 | 2 | 2 | 2 | 3 |
| L3-0609 | *B. abortus* | 2 | 3 | 3 | 3 | 4 |
| L3-0633 | *B. abortus* | 2 | 3 | 3 | 3 | 4 |
| L3-0635 | *B. abortus* | 2 | 3 | 3 | 3 | 4 |
| L3-0638 | *B. abortus* | 2 | 3 | 3 | 3 | 4 |
| L3-2518 | *B. abortus* | 2 | 3 | 3 | 3 | 4 |
| L3-2519 | *B. abortus* | 2 | 3 | 3 | 3 | 4 |
| L3-2522 | *B. abortus* | 2 | 3 | 3 | 3 | 4 |
| L3-2523 | *B. abortus* | 2 | 3 | 3 | 3 | 4 |
| L3-2525 | *B. abortus* | 2 | 3 | 3 | 3 | 4 |
| L3-2526 | *B. suis* | 1 | 1 | 1 | 1 | 1 |
| L3-2527 | *B. suis* | 1 | 1 | 1 | 1 | 2 |
| L3-2528 | *B. suis* | 1 | 1 | 1 | 1 | 2 |
| L3-2529 | *B. suis* | 1 | 1 | 1 | 1 | 1 |
| L3-2531 | *B. suis* | 1 | 1 | 1 | 1 | 1 |
| L3-3287 | *B. melitensis* | 3 | 4 | 4 | 4 | 5 |
| L3-4054 | *B. suis* | 1 | 1 | 1 | 1 | 2 |
| L3-4547 | *B. suis* | 1 | 1 | 1 | 5 | 6 |
| L3-4552 | *B. melitensis* | 3 | 4 | 4 | 4 | 5 |
| L3-4553 | *B. melitensis* | 3 | 4 | 4 | 4 | 5 |
| L3-4554 | *B. melitensis* | 3 | 4 | 4 | 4 | 5 |

| | | | | | | |
|---|---|---|---|---|---|---|
| L3-4556 | *B. melitensis* | 3 | 4 | 4 | 4 | 5 |
| L3-4557 | *B. melitensis* | 3 | 4 | 4 | 4 | 5 |
| L3-4558 | *B. melitensis* | 3 | 4 | 4 | 4 | 5 |
| L3-4559 | *B. melitensis* | 3 | 4 | 4 | 4 | 5 |
| L3-4560 | *B. melitensis* | 3 | 4 | 4 | 4 | 5 |
| L3-4561 | *B. melitensis* | 3 | 4 | 4 | 4 | 5 |
| L3-4562 | *B. melitensis* | 3 | 4 | 4 | 4 | 5 |
| L3-4563 | *B. melitensis* | 3 | 4 | 4 | 4 | 5 |
| L3-4564 | *B. melitensis* | 3 | 4 | 4 | 4 | 5 |
| L3-4565 | *B. melitensis* | 3 | 4 | 4 | 4 | 5 |
| L3-4579 | *B. melitensis* | 3 | 4 | 4 | 4 | 5 |
| L3-4588 | *B. melitensis* | 3 | 4 | 5 | 6 | 7 |
| L3-4589 | *B. melitensis* | 3 | 4 | 4 | 4 | 5 |
| L3-4590 | *B. melitensis* | 3 | 4 | 4 | 4 | 5 |
| L3-4599 | *B. melitensis* | 3 | 4 | 4 | 4 | 5 |
| L3-4602 | *B. melitensis* | 3 | 4 | 4 | 4 | 5 |

Table S8: Confusion matrix showing the true performance of fine-tuned NN model when applied on to the external dataset, encompassing 12 *Brucella* sp. The model was trained on 41 samples using three most important features, namely Peak.6715, .8324 and .9863. The diagonal elements of the confusion matrix indicate correct predictions, while the offdiagonals represent incorrect prediction. The classifier was modelled with the following tune parameters: size=3 and a decay=0. The samples L3-2519 *B. abortus* and L3-4602 *B. melitensis* were both incorrectly classified as *B. suis*.

| | **NN model prediction / classification** | | | |
|---|---|---|---|---|
| | | ***B. abortus*** | ***B. melitensis*** | ***B. suis*** |
| **True class** | ***B. abortus*** | 2 | 0 | 0 |
| | ***B. melitensis*** | 0 | 5 | 0 |
| | ***B. suis*** | 1 | 1 | 3 |
| | Overall Accuracy: 0.83 | | | |
