# Supplementary figures and images for: Machine Learning Algorithms for Classification of MALDI-TOF MS Spectra from Phylogenetically Closely Related Species Brucella melitensis, Brucella abortus and Brucella suis
Source: Microorganisms. 2022 Aug 17;10(8):1658. doi: 10.3390/microorganisms10081658 (PMC9416640; doi:10.3390/microorganisms10081658)

**Figure S2:** Box-plot displaying Peak.6715 for the Kruskal-Wallis and Wilcoxon tests.

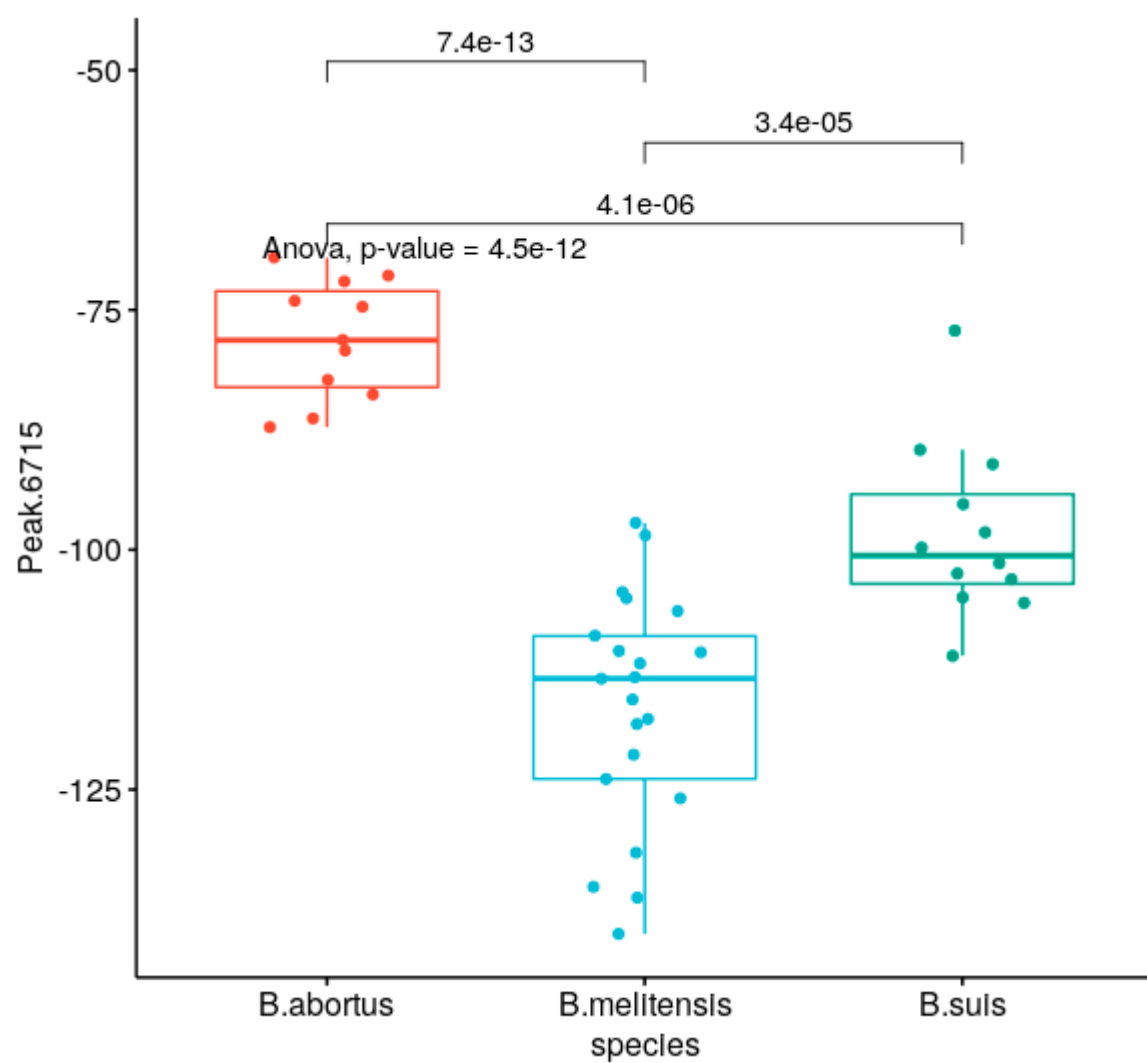

Supplement: Supplementary file 1 [file microorganisms-10-01658-s001.zip › Figure S2.pdf]

**Figure S5:** Average silhouette coefficient vs. different distance-linkage combinations.

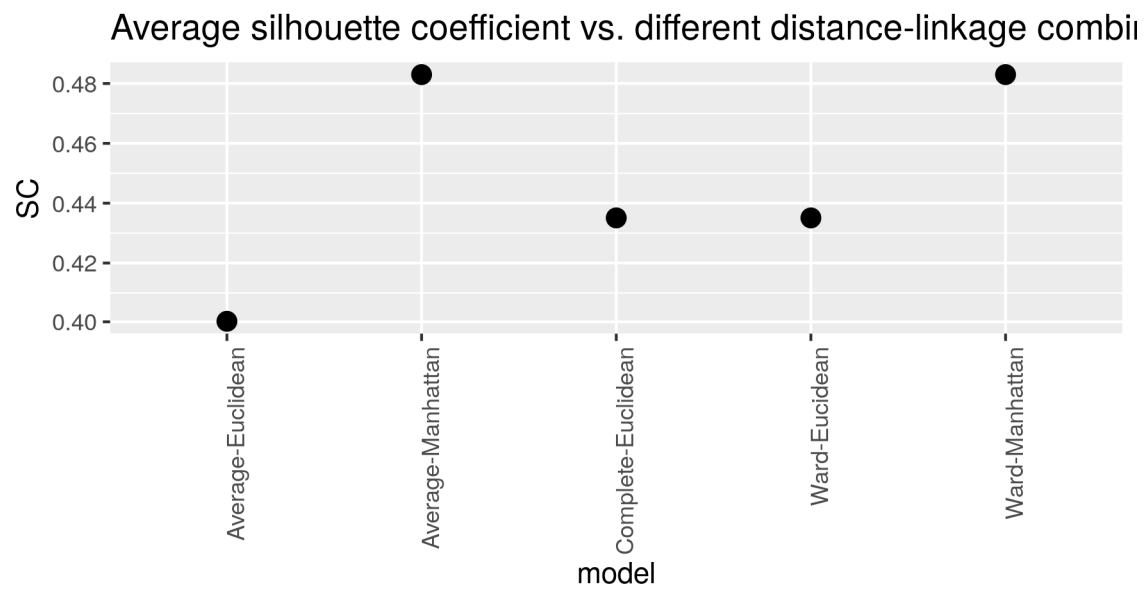

Supplement: Supplementary file 1 [file microorganisms-10-01658-s001.zip › Figure S5.pdf]
